# Artesunate-induced hemolysis in severe complicated malaria – A diagnostic challenge: A case report and literature review of anemia in malaria

**DOI:** 10.1016/j.idcr.2021.e01234

**Published:** 2021-07-24

**Authors:** Sundus Sardar, Mohammed Abdurabu, Ahmed Abdelhadi, Mhd Baraa Habib, Muhammad Bilal Jamshaid, Adnan Humam Hajjar, Munir Abu Ageila, Tasnim Abdalla, Anand Kartha, Khalid Farooqui

**Affiliations:** a Department of Internal Medicine, Hamad Medical Corporation, Doha, Qatar; b Department of Emergency Medicine, Hamad Medical Corporation, Doha, Qatar; c College of Medicine, Qatar University, Doha, Qatar

**Keywords:** Malaria, Anemia, Post-artemisinin delayed hemolysis, PADH, *Plasmodium falciparum*

## Abstract

Malaria infection, which results due to the parasitic protozoan *Plasmodium*, has several known etiologies of hemolytic anemia as a possible complication in cases such as concurrent G6PD deficiency, severe parasitemia, or use of parenteral antimalarials. Although artemisinin-based antimalarial therapies are generally well-tolerated, several cases of severe post-artemisinin delayed hemolysis (PADH) have been recently reported, which present a diagnostic challenge, and affect morbidity and mortality in patients with malarial infection. We highlight the case of a young lady with *Plasmodium falciparum* severe parasitemia who developed hemolytic anemia after parenteral artesunate therapy.

## Introduction

Malaria is a major health problem worldwide. It results from infection by parasitic protozoans belonging to the genus *Plasmodium.* The *Plasmodium falciparum* and *Plasmodium vivax* cause the majority of human malarial infections, with *P. falciparum* being the more virulent [1].

Malaria affects many countries, especially those with poor public health resources, and it has been reported that the incidence of the disease in 2004 was between 350 and 500 million cases. Over two billion people, representing more than 40 % of the world's population, are at risk of contracting malaria, and the number of deaths due to malaria worldwide has been estimated at 1.1–1.3 million annually in reports by World Health Organization (WHO) between 1999–2004 [2].

Malaria typically presents with high fever, which may be accompanied by chills and headache. Symptoms and signs may be generally be more subtle, and may also include anemia and hepatosplenomegaly. Patients with malaria may also experience respiratory distress and/or rapidly progressing cerebral malaria, manifesting as altered sensorium and, infrequently, as seizures. For initial diagnosis, thick blood smears are performed; however, a single smear showing no evidence of parasites is not sufficient to exclude malarial infection. To identify the type of parasitic species, thin blood smears are used [3]. Management involves initiation of anti-malarial drugs as per treatment guidelines such as artesunate, quinine or mefloquine, etc., alongside supportive measures.

The parenteral artemisinin drug, artesunate, is a first-line treatment for severe *Plasmodium falciparum* infection worldwide, and has resulted in saving more patients with severe malaria than the previous reference drug quinine. Use of artesunate is associated with fatality rates consistently lower than 5%; however, a considerable proportion of patients are affected by hemolytic episodes occurring after parasitological cure. Such episodes are referred to as post-artemisinin delayed hemolysis (PADH) [4].

PADH is linked to the destruction of circulating infected red blood cells (i-RBCs) from which, after artesunate therapy parasites have been removed by “pitting” in the spleen, thereby deemed as once-infected RBCs (O-iRBCs) [4].

Here, we present this rare case of post-artesunate delayed hemolysis to highlight diagnostic challenges, to improve patient care, management plan and result in better treatment outcomes.

## Case presentation

We present the case of a previously healthy 35-year-old female, who presented to our emergency department (ED) complaining of high-grade fever of waxing-and-waning nature for five days, associated with vomiting, body aches and sleepiness. She works at a local bank and developed fever during her mandated hotel quarantine upon return from a holiday in Sudan seven days ago, during which she was not on malaria prophylaxis. COVID-19 PCR from nasopharyngeal swab was negative. She did not improve despite anti-pyretics and anti-emetics during the hotel quarantine, and sought medical advice at a private clinic where she tested positive for malaria and was referred to our hospital for inpatient management.

Patient reported that she had previous history of malaria 10 years ago but not as severe. She had no other sick contact in her family or friends.

Her initial labs showed hemoglobin of 12.1 gm/dl, normal white blood cell count of and platelets of 150 with elevated AST and ALT of 58 and 57U/L, respectively and high total bilirubin of 26 μmol/L. Malarial smears were positive for *Plasmodium falciparum* with a parasitemia level of 27 %. She was admitted to medical intensive care unit (MICU) for further management under close monitoring due to severe malaria with overlapping features of sepsis and acute respiratory distress syndrome (ARDS). In line with guidelines for malaria management, she was started on parenteral artesunate, which was administered until the parasite level decreased to less than 1%. She was then started on oral artemether-lumefantrine for five days thereafter, and the malaria parasite level at the end of the treatment was less than 0.1 %. In the meantime, she received other supportive treatment including intravenous fluids, antiemetics, antipyretics and antibiotics.

She was transferred to medical ward after MICU care, and had significant improvement in her condition, except for persistent fever and a gradual decline in her hemoglobin.

**Fig. 1:**
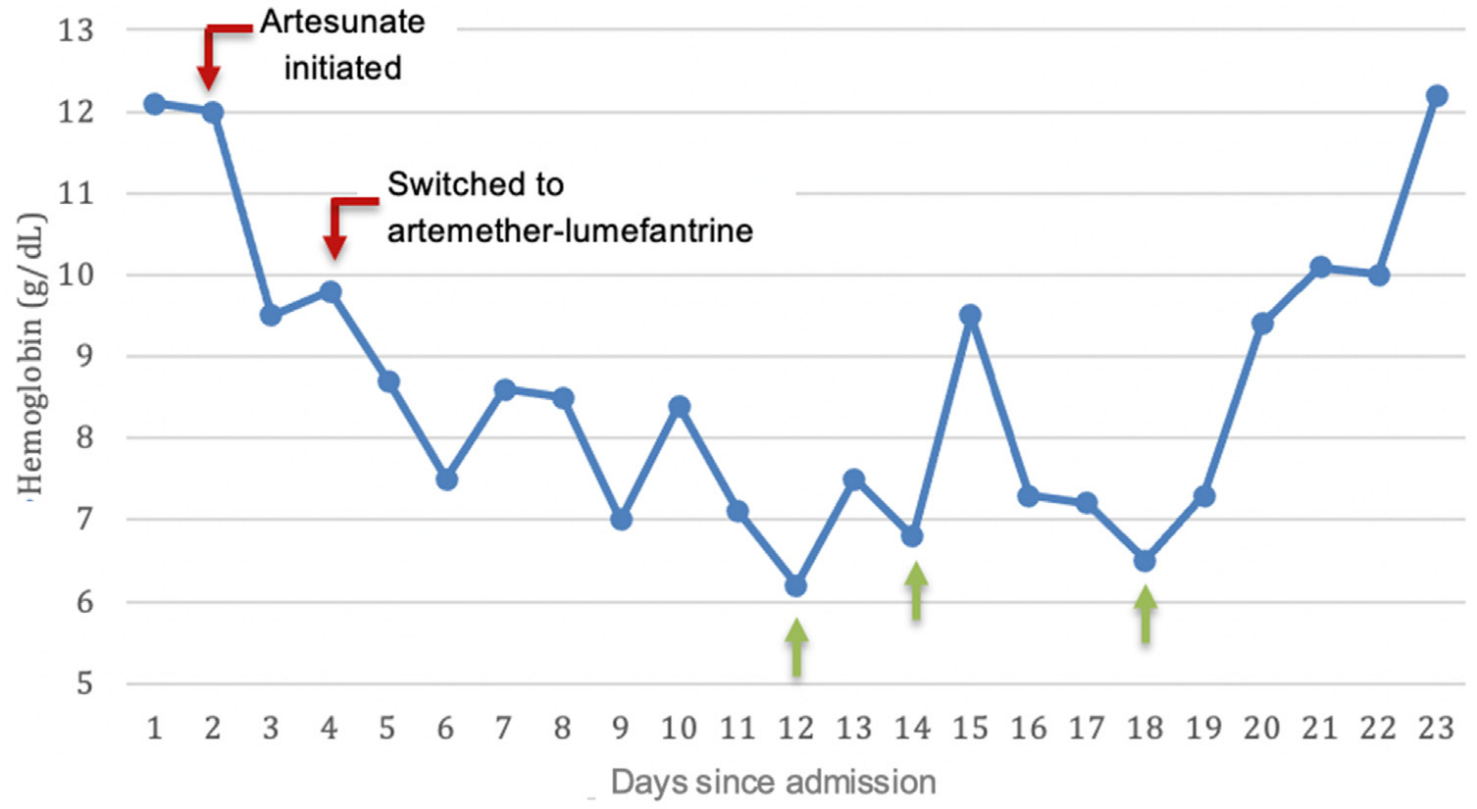
Hemoglobin trend during hospitalization with intravenous artesunate therapy followed by artemether-lumefantrine; with hemoglobin level < 7 g/dL at three instances during hospital admission, requiring packed red blood cell transfusions (green arrows).

Labs later in the hospital course revealed continuous gradual drop in hemoglobin from 12 g/dL to 6.5 g/dL and she received multiple packed red blood cell transfusions. Her hemoglobin would initially increase post-transfusions, followed by a decrease again within one to two days. Figure 1 shows the trend of hemoglobin levels during the hospitalization along with blood transfusions throughout the admission. Later in her clinical course, her platelets dropped from 160 to 70 × 10^3^/μL, but improved without intervention. Liver transaminases also increased up to 150sU/L and total bilirubin was 35 μmol/L. Labs revealed increased lactate dehydrogenase (LDH) of 595 U/L (reference range: 135–214 U/L) and low haptoglobin reported as less than 10 mg/dl (30−200 mg/dl). Coombs’ test was positive with low complement 4 (C4). Subsequently, all liver function tests improved within one week.

She continued to spike fever at least once per day and temperatures ranged from 38.5 to 40C and she was on as-needed paracetamol infusion. Sepsis workup including blood cultures and urine culture were negative. She had bilateral pleural effusion, more prominent on the right side associated with dyspnea and increased requirement for oxygen supplementation, thus piperacillin-tazobactam was initiated empirically and she underwent therapeutic and diagnostic thoracentesis on the left side – as the right-sided effusion was complicated with consolidation and collapse. Pleural fluid analysis revealed exudative effusion with predominantly neutrophilic leukocytosis.

In the next few days, she continued to spike fever and had persistent drop in hemoglobin, due to which antimicrobial coverage was escalated to tigecycline to treat suspected empyema, for which right-sided effusion was drained by interventional radiologist, and pleural fluid analysis showed the same results as those from left pleural effusion. Once the results of all cultures from pleural fluid and peripheral blood were negative, the antibiotics were discontinued.

**Fig. 2:**
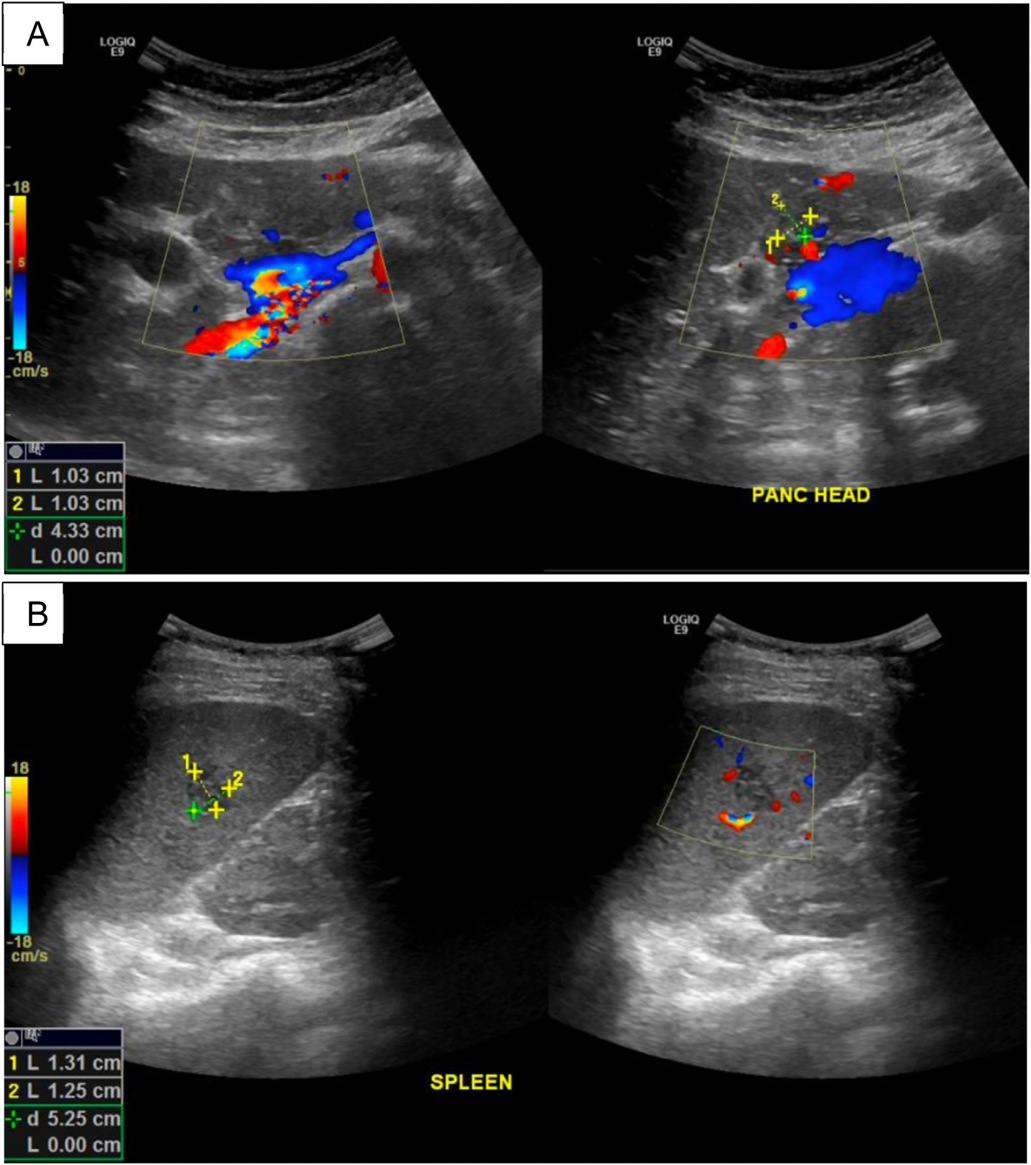
Ultrasound of the abdomen showing ill-defined hypoechogenic lesions in pancreatic head (A) and spleen (B).

Despite all measures, there was no further improvement in her fever. Further workup to investigate possible fever of unknown origin included ultrasound of the abdomen to evaluate for any intrabdominal collections and it showed an ill-defined hypoechogenicity in the pancreatic head (Fig. 2) and multiple ill-defined hypoechoic lesions in the spleen and recommended for further evaluation by magnetic resonance imaging (MRI). The ultrasound also incidentally noted multiple gallbladder polyps, minimal pelvic free fluid and bilateral pleural effusion. Contrast-enhanced MRI of the abdomen confirmed multiple splenic infarcts, mildly enlarged liver and annular pancreas with no focal pancreatic lesion.

As no significant improvement was noted in her clinical condition, further workup was performed to exclude connective tissue disease, involving ANA which was positive. Direct antiglobulin test was also positive, along with high LDH and low haptoglobin, confirming autoimmune hemolytic anemia. Bone marrow aspiration (BMA) was not performed as the patient declined the procedure after discussion of benefits of BMA in diagnosis and potential risks of the procedure including hematoma and bleeding, especially considering thrombocytopenia. After ruling out all other causes including hemolytic anemia due to autoimmune etiologies or related to sepsis, along with resolution of the fever, the delayed autoimmune hemolytic anemia was deemed most likely attributable to parenteral artesunate in our case.

After one-week follow-up, she had stable hemoglobin and her fever chart showed no more spikes with good over all condition as per the patient.

As per our conclusions, with accumulating available information, it appears our patient had a rare case of artesunate-induced delayed hemolysis by exclusion of all other possible diagnoses and we highlight the challenges of diagnosis of PADH due to its rare occurrence and as the concept is not yet well-recognized.

## Discussion

Anemia is a common complication of severe malaria and it can be caused by the parasite or the antimalaria medications [5,6]. Malaria infection causes hemolytic anemia by two main mechanisms: immune and non-immune mediated hemolysis. The main cause is non-immune, which results from direct destruction of the red blood cells (RBCs) by the parasite, and depends on the type of malaria and the percentage of parasitemia [7]. It usually occurs in patients with severe malaria, which is most commonly caused by *Plasmodium falciparum*. Intravenous artesunate is the recommended parenteral therapy by WHO for severe malaria [8] as studies have shown that it decreases mortality and morbidity [9]. Although recently, it has been reported that hemolysis is one of its common side effects [10].

The mechanism of artesunate-induced hemolysis, referred to as post-artemisinin delayed hemolysis (PADH), is not fully understood but it is speculated to be secondary to the destruction of infected RBCs after killing the parasite and usually occurs anytime between few days to four weeks after receiving the medication [10]. Thus, it is recommended to follow the hemoglobin level 7, 14, and 30 days after treatment [11,12]. Some previously speculated PADH to be associated with specific drug formulations, while other evidence refutes this possible hypothesis [6,13,14].

**Fig. 3:**
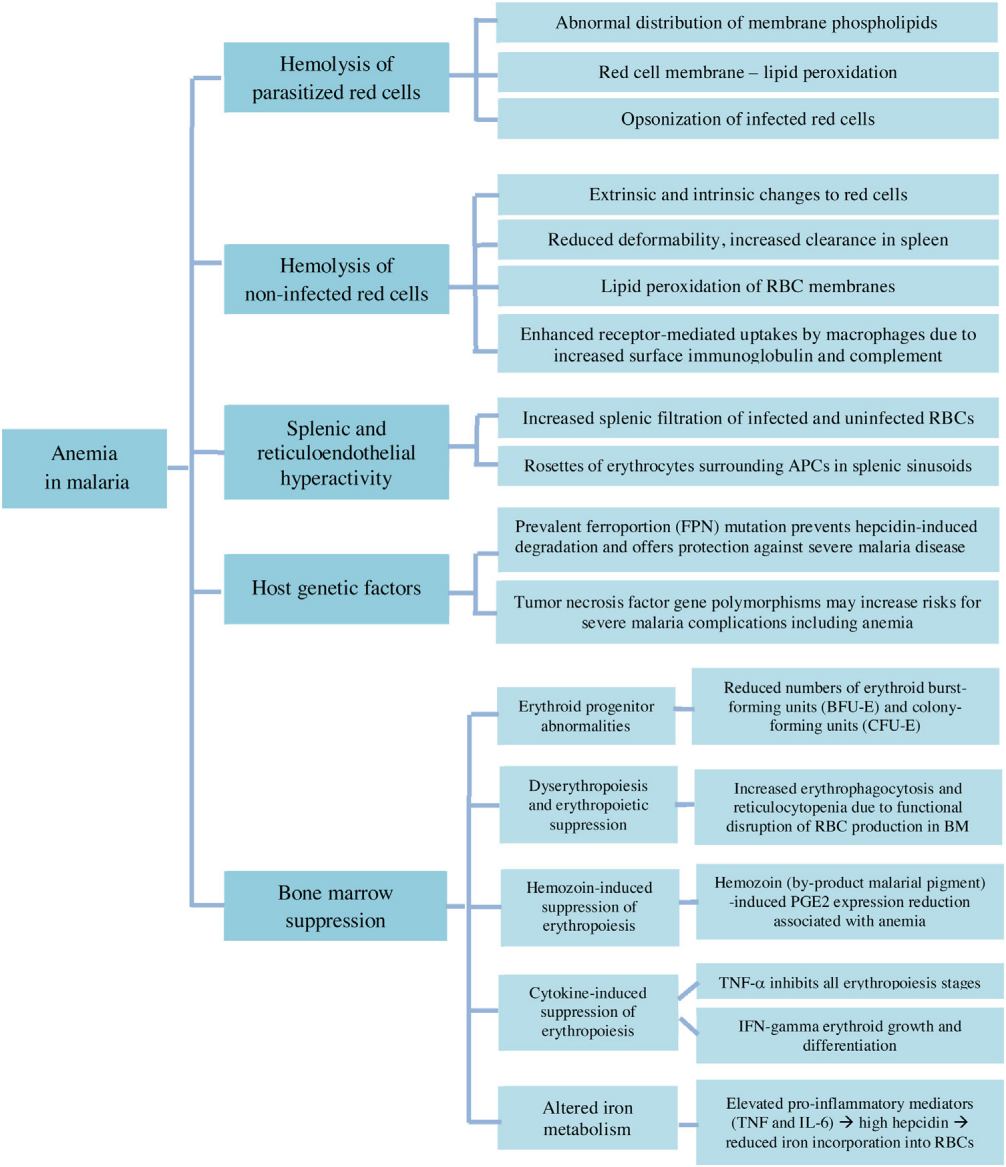
**An overview of etiologic factors for anemia in malaria**. RBC: Red blood cells; APCs: antigen-presenting cells; BM: bone marrow; PGE2: Prostaglandin E2; TNF-α: Tumor necrosis factor-alpha; IFN: Interferon; IL-6: Interleukin-6.

While the etiology of anemia in malaria is multifactorial (Fig. 3), autoimmune hemolytic anemia is rarely associated with malaria and, so far, it has been documented in few case reports and case series [15,16]. Also, it is infrequently caused by artesunate [17]. Based on this information, it might be difficult to determine the cause of hemolysis in a patient with severe malaria.

Our patient was admitted as a case of severe *Plasmodium falciparum* malaria with parasitemia level of 23 %. She received three doses of IV artesunate and showed good clinical response along with her reduction in parasitemia level to less than 0.1 % after 3 days. Despite this improvement, she continued to produce dark urine, her hemoglobin level declined, LDH increased and haptoglobin decreased, and moreover, Coombs’ test was positive with low complement 4 (C4). She improved after packed red blood cell transfusion. After few days, her hemoglobin dropped again and responded to blood transfusion once more. Coombs’ test repeated after 1 week was positive again. Further workup to exclude other underlying causes such as connective tissue disease and lymphoma were all negative. Her case was thoroughly discussed with the hematologist, who recommended supportive management as she had completed her antimalarial therapy course and no other clear causes of hemolytic anemia were identified.

With supportive measures, her hemoglobin level improved, and she was thus discharged with outpatient clinic follow-up after four weeks, upon which she had improved hemoglobin and stable but low C4.

We can conclude that this uncommon presentation and its diagnosis is a challenging feat for physicians to suspect clinically and exclude other possible etiologies, along with prolonged hospital course and sometimes, extensive investigations. The authors would like to emphasize the important key points in this case for physicians to be aware of this subtype of hemolytic anemia associated with intravenous artesunate therapy in patients managed for malaria, which thereby requires close observation and careful supportive management.

## Conclusion

Post-artemisinin delayed hemolysis (PADH) is a rare cause of prolonged autoimmune hemolysis in patients with severe *Plasmodium falciparum* malaria. Blood transfusions, supportive measures and close follow-ups are helpful in appropriate management; however, more studies are required to ascertain the demographics, possible predictors and associated factors of PADH for a better understanding of the concept to facilitate diagnosis and curb morbidity and mortality.

## Funding

Qatar National library funded the open access publication fees of this case.

## Ethical approval

Obtained for publication by local medical research committee.

## Consent

Written informed consent was obtained from the patient for publication of this case report.

## Author contributions

Writing the initial draft of the manuscript: Sundus Sardar, Mohammed Abdurabu.

Conceptualization and supervision: Anand Kartha, Khalid Farooqui, Sundus Sardar.

Medical management of the case: Anand Kartha, Mohammed Abdurabu, Sardar Sundus, Ahmed Abdelhadi, Muhammad Bilal Jamshaid, Khalid Farooqui.

Additions to manuscript: Adnan Humam Hajjar, Munir Abu Ageila, Tasnim Abdalla, Mhd Baraa Habib.

Revising the manuscript critically and literature review: Sundus Sardar, Khalid Farooqui.

The first authors (SS and MA) contributed equally to the writing and preparation of this article. SS, MA, AA have written the initial draft of the manuscript and performed the literature review. The draft was revised and updated by SS with supervision from AK and KF. The authors AK, SS, MA, AA, MBJ, AHH, MAA, TA, MBH, KF were all part of the medical treating team and submitted additions to the manuscripts. All the authors critically reviewed the initial and the final draft of the manuscript and approved it for submission.

## Declaration of Competing Interest

The authors report no declarations of interest.
